# Ketamine-propofol (Ketofol) for procedural sedation and analgesia in children: a systematic review and meta-analysis

**DOI:** 10.1186/s12873-020-00373-4

**Published:** 2020-10-08

**Authors:** Tze Yong Foo, Norhayati Mohd Noor, Mohd Boniami Yazid, Mohd Hashairi Fauzi, Shaik Farid Abdull Wahab, Mohammad Zikri Ahmad

**Affiliations:** 1 Department of Emergency Medicine, Universiti Sains Malaysia, School of Medical Sciences, Kubang Kerian, Malaysia; 2 Department of Family Medicine, Universiti Sains Malaysia, School of Medical Sciences, Kubang Kerian, Malaysia; 3 Hospital Universiti Sains Malaysia, Kubang Kerian, Malaysia

**Keywords:** Children, Paediatric, Ketofol, Ketamine-propofol, Procedural sedation and analgesia, Systematic review, Meta-analysis

## Abstract

**Objectives:**

The aim of this review is to elucidate the efficacy and side effects of ketofol in comparison to other anaesthetic agents during procedural sedation and analgesia.

**Method:**

The Cochrane Central Register of Controlled Trials (1996 to Feb 2019) and MEDLINE (1966 to Feb 2019) were searched, including the related randomised control trials and reviewed articles to find unpublished trials or trials not obtained via electronic searches. Inclusion criteria for the studies included comparing recovery time, recording clinician satisfaction, and assessing the adverse effects of ketofol.

**Results:**

Eleven trials consisting of a total of 1274 patients met our criteria and were included in this meta-analysis. Five trials compared ketofol with a single agent, while six trials compared ketofol with combined agents. While comparing between ketofol and a single agent (either ketamine or propofol), ketofol showed significant effect on recovery time (MD: -9.88, 95% CI: − 14.30 to − 5.46; *P* = 0.0003; I^2^ = 92%). However, no significant difference was observed while comparing ketofol with combined agents (RR: 0.75, 95% CI: − 6.24 to 7.74; *P* < 0.001; I^2^ = 98%). During single-agent comparison, ketofol showed no significant differences in terms of clinician satisfaction (RR: 2.86, 95% CI: 0.64 to 12.69; *P* = 0.001; I^2^ = 90%), airway obstruction (RR: 0.72, 95% CI: 0.35 to 11.48; *P* = 0.81; I^2^ = 0%), apnoea (RR: 0.9, 95% CI: 0.33 to 2.44; *P* = 0.88; I^2^ = 0%), desaturation (RR: 1.11, 95% CI: 0.64 to 1.94; *P* = 0.28; I^2^ = 21%), nausea (RR: 0.52, 95% CI: 0.91 to 1.41; *P* = 0.2; I^2^ = 38%), and vomiting (RR: 0.63, 95% CI: 0.25 to 1.61; *P* = 0.18; I^2^ = 42%). During comparison with combined agents, ketofol was more effective in reducing hypotension (RR: 4.2, 95% CI: 0.2 to 0.85; *P* = 0.76; I^2^ = 0%), but no differences were observed in terms of bradycardia (RR: 0.70, 95% CI: 0.14 to 03.63; *P* = 0.09; I^2^ = 53%), desaturation (RR: 1.9, 95% CI: 0.15 to 23.6; *P* = 0.11; I^2^ = 61%), and respiratory depression (RR: 1.98, 95% CI: 0.18 to 21.94; *P* = 0.12; I^2^ = 59%).

**Conclusion:**

There is low certainty of evidence that ketofol improves recovery time and moderate certainty of evidence that it reduces the frequency of hypotension. There was no significant difference in terms of other adverse effects when compared to other either single or combined agents.

**Trial registration:**

PROSPERO CRD42019127278.

## Background

Procedural sedation and analgesia (PSA) is a treatment strategy involving the administration of agents with sedative, analgesic, or dissociative properties to suppress a patient’s consciousness to varying degrees. It facilitates the completion of painful procedures, while ensuring the safety and comfort of the patient without compromising airway patentability [1]. The demand of PSA outside of the operation theatre (OT) has grown exponentially for both diagnostic and therapeutic purposes, especially in the emergency department (ED) and intensive care unit (ICU) [2], as the clinician develops confidence in managing the sedated patients, especially the paediatric population [3]. Various types of unpleasant procedures require optimal sedation and analgesia in order to increase the procedure’s success rate, clinician satisfaction, and to reduce the discomfort and anxiety of the patients, especially the paediatric population. As a standard of healthcare, the American College of Emergency Physicians recommends effective management of anxiety and discomfort associated with such procedures [1]. In an era of increased emphasis on maintaining quality and minimising costs, PSA outside the OT always offers significant advantages in terms of delivering timely and cost-effective care to the patient [4].

Ideal PSA agents should have predictable effects on the regulation of the sedation, analgesia, amnesia, and motor function. In addition, it is beneficial to administer an agent with fast induction, steady control, quick recovery time, and minimal side-effects. Unfortunately, despite the availability of numerous anaesthetic agents, no single agent demonstrates superiority over the others. Thus, various combinations of different sedative, analgesic, or dissociative agents have been considered in order to optimise the desired effect and minimise the adverse effect [5]. However, PSA in the paediatric population remains a challenge, as the sedation risk is higher in children [3]. The anatomical and physiological differences between the paediatric and adult airways make it potentially more difficult and complicated to manage the former. Medical problems affecting the airway, breathing, circulation, and neurological function play an important role in increasing the risk of PSA. The Pediatric Sedation Research Consortium (http://www.pedsedation.org) in the United States of America (USA) is a multi-centre registry involving over 30 centres that practice PSA [6]. It reported that there were a total of 1020 adverse events over 30,000 PSA procedures undertaken outside OT with no death, one cardiac arrest, and required cardiopulmonary resuscitation. Less serious events were more commonly associated with desaturation, which occurring 157 times over 10,000 sedation procedures. Unexpected apnoea, excessive secretions, and vomiting had frequencies of 24.0, 41.6, and 47.2 per 10,000 procedures, respectively [7].

A large number of ED in the USA have adopted ketofol as their primary sedation regime [8]. Both drugs exhibit a synergistic reaction to each other and theoretically offset each other’s adverse effects. A combination of the drugs allows a smaller dose of each one, thus potentially improving the quality, safety, and duration of recovery time [9]. A recent study had shown that ketofol reduces complications in PSA in adults compared to propofol alone [5]. But the superiority and safety of ketofol in the paediatric population are still debatable. If it is proven to be a safe and effective anaesthetic agent in PSA in children, it should be considered as a primary choice. This study was conducted to elucidate the efficacy and role of ketofol in comparison to other anaesthetic agents in PSA in paediatric patients.

## Method

The Cochrane Central Register of Controlled Trials (CENTRAL) (1996 to Feb 2019) and the Medical Literature Analysis and Retrieval System Online (MEDLINE) (1966 to Feb 2019) were searched for relevant literature. We used the search strategy in Appendix for CENTRAL and MEDLINE searches. We restricted to the publications in English language. We searched the reference list of known randomised control trials (RCTs) and reviewed papers to identify unpublished trials or trials that are not found by electronic searches. We searched the World Health Organization International Clinical Trials Registry Platform (ICTRP) (http:/www.who.int/ictrp/en/) and www.clinicaltrials.gov for on-going trials.

All the RCTs or clinical control trials comparing ketofol with other anaesthetic agents were included in the study. We included blinded and open-labelled studies including patients below 18 years old who underwent procedural sedation. The primary outcomes were recovery time and satisfaction of the clinician. Secondary outcomes included adverse events (such as nausea, vomiting, airway obstruction, apnoea, desaturation, respiratory depression, hypotension, and bradycardia) and hemodynamic parameters (heart rate, respiratory rate, and blood pressure). The clinical outcomes were defined based on the assessment of the managing clinicians.

We evaluated the search titles and abstracts and accessed full-text articles when they appeared to meet the eligibility requirements, or when the title or the abstract provided insufficient information to determine the eligibility. We independently assessed the validity of the trials and reported the reasons for the exclusion. We resolved any disputes through discussions between the review authors. If clarification was required, we contacted the authors of the publications. Using data extraction form, from each of the selected trials, we extracted data on study setting, participant characteristics (age, sex, and ethnicity), methodology (number of participants randomised and analysed), type and dosage of anaesthetic agent, recovery time, clinicians’ and parents’ satisfaction, the occurrence of related adverse events, and hemodynamic parameters, independently.

We evaluated the parameters of risk of bias, such as random sequence generation, allocation concealment, blinding of participants and personnel, blinding of outcome assessors, completeness of data, selectivity of outcome reporting, and other biases [10]. We performed the risk of bias assessment independently and resolved any disagreements by discussion. We assessed the quality of evidence for primary and secondary outcomes according to the Grading of Recommendations Assessment, Development and Evaluation (GRADE) methodology [11] for risk of bias, inconsistency, indirectness, imprecision, and publication bias; classified as very low, low, moderate, or high.

We measured the treatment effect for dichotomous outcomes using risk ratios (RRs) and absolute risk reduction, and for continuous outcomes, we used mean differences (MDs), both with 95% confidence intervals (CIs). We reported the results of the random-effects model. We examined for a unit of analysis errors on the included trials. Unit of analysis errors can occur when trials randomise participants to intervention or control groups in clusters but analysed the results using the total number of individual participants. We adapted the test results showing a unit of analysis errors based on the mean cluster size and coefficient of the intracluster correlation, if any [10]. We intended to contact the original authors of the trials to request missing or improperly reported data. If missing data were not available, we analysed the available data. We undertook meta-analyses using Review Manager (RevMan) version 5.3.5 (Nordic Cochrane Centre, Cochrane Collaboration). In two measures, we tested the existence of the heterogeneity. First, through examining populations, conditions, interventions and results, we measured the apparent heterogeneity at face value. Second, we analysed statistical heterogeneity using the statistic I^2^. We used the heterogeneity analysis guide, as follows: May not be significant between 0 and 40%, moderate heterogeneity between 30 and 60%; substantial heterogeneity between 50 and 90%, and considerable heterogeneity between 75 and 100% [10]. If there were enough trials, we intended to use a funnel plot to explore the possibility of reporting biases or small trial biases, or both. We conducted a sensitivity analysis to analyse the influence of bias risk for sequence generation and allocation concealment.

## Result

**Fig. 1:**
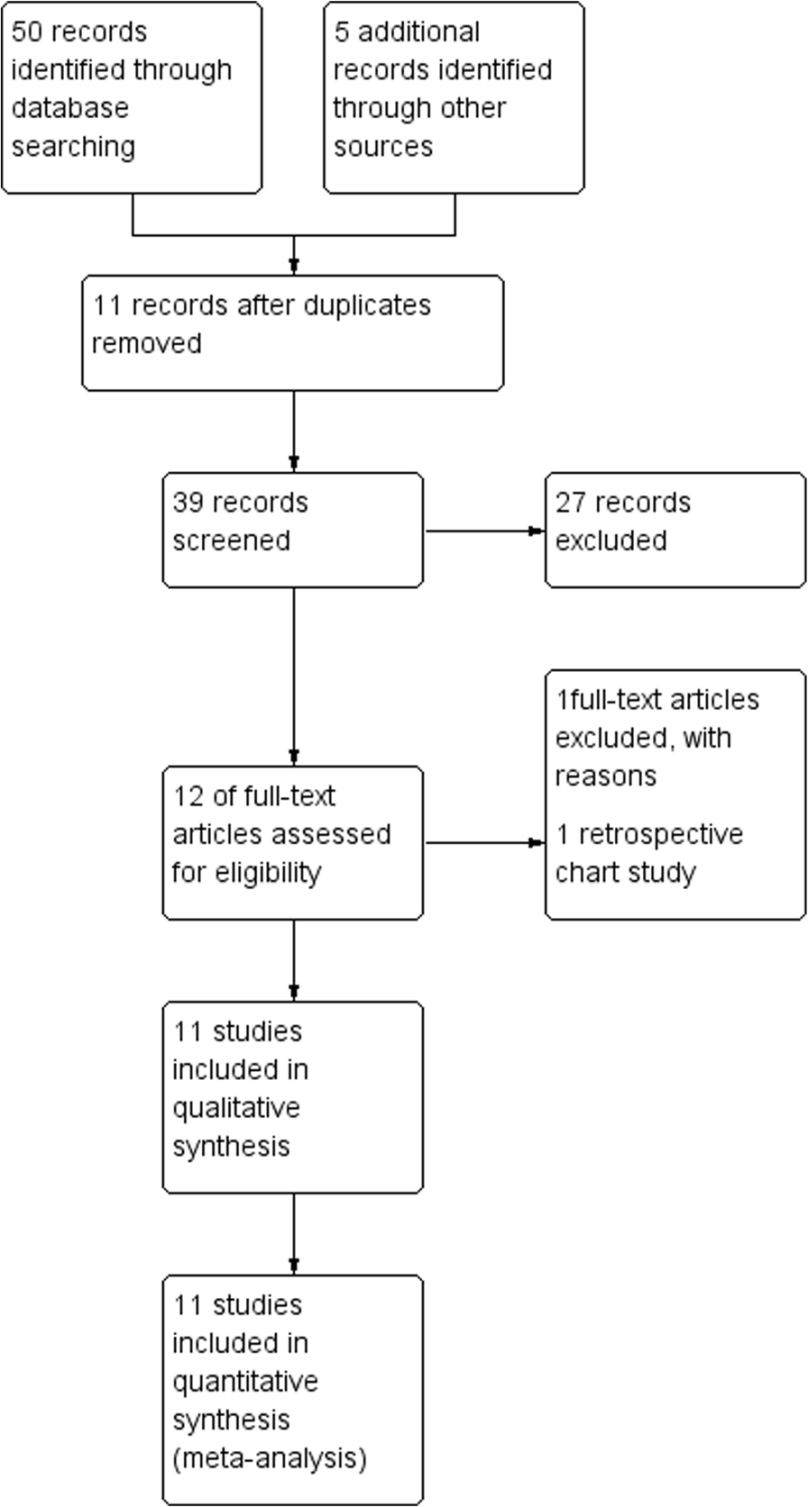
PRISMA flow chart

We retrieved 50 potentially relevant studies from the search of the electronic databases. After excluding duplicate results and those that did not meet the eligibility criteria, we reviewed 12 full-text articles. One study was excluded due to non-RCT [12]. Finally, 11 RCTs met the review eligibility criteria [13–23] (Fig. 1).

We included 11 trials with a total of 1274 patients. All trials were single-centre studies. Four trials included 121 to 351 patients [13, 15, 17, 18], while seven trials included 46 to 92 patients [14, 16, 19–23]. The age of the patients ranged from 1 month to 16 years old. The trials involved three orthopaedic and one surgical procedures [14, 17–19], five invasive procedures [13, 16, 20–22], one elective imaging study [15], and one dental procedure [23]. Table 1 summarises the characteristics of included trials.

**Table 1:** Characteristics of included trials

Study	Intervention	Dose of ketofol	Dose of control	n	Age	Procedure	Outcome
Canpolat 2012 [19]	Ketofol vs. ketamine-dexmedetomidine	● 2 mL ketamine (50 mg/mL) + 8 mL NS ● 1 mg/kg propofol followed by 1 mg/kg ketamine ● Additional 1 mg/kg propofol, if needed	● 2 mL ketamine (50 mg/mL) + 8 mL NS ● 0.5 mL dexmedetomidine (50 μg) + 9.5 mL NS ● 0.5 μg/kg dexmedetomidine followed by 1 mg/kg ketamine ● Additional 0.5 μg/kg dexmedetomidine, if needed	60	8 months to 5 years	Burn injury dressing	1. Surgeon satisfaction 2. Adverse effects 3. Hemodynamic parameters
Chiaretti 2011 [13]	Ketofol vs. propofol	● 0.5 mg/kg ketamine before propofol injection ● 2 mg/kg propofol over 2 min ● 0.5–1 mg/kg additional dose, if required	● 2 mg/kg bolus over 2 min ● 0.5–1 mg/kg additional dose, if needed	121	Ketofol: mean (SD): 6.9 (5.4) years Propofol: mean (SD): 7.3 (5.2) years	Lumbar puncture or bone marrow aspiration	1. Adverse effects 2. Hemodynamic parameters
Joshi 2017 [20]	Ketofol vs. dexmedetomidine-ketamine	● 1 mg/kg propofol, 1 mg/kg ketamine ● Maintenance IV infusion of 100 μg/kg/min of propofol and 1 mg/kg/h of ketamine ● Additional 0.5 mg/kg ketamine, if needed	● 1 μg/kg dexmedetomidine IV infusion over 1 min + 1 mg/kg ketamine IV bolus ● Maintenance IV infusion of 0.5 μg/kg/h of dexmedetomidine and 1 mg/kg/h of ketamine ● Additional 0.5 mg/kg ketamine, if needed	60	1 month to 6 years	Cardiac catheterisation procedure	1. Recovery time 2. Hemodynamic parameters
Khutia 2012 [14]	Ketofol vs. propofol-fentanyl	● Ratio 1:2 mixing 1 mL ketamine (50 mg/mL) with 10 mL of 1% propofol (10 mg/mL) ● Each mL contains 9 mg propofol: 4.5 mg ketamine ● Bolus: 1 mg/kg propofol, 0.5 mg/kg ketamine ● Infusion of 50 μg/kg/min	● 10 mL of 1% propofol mixed with 1 mL NS (9 mg/mL) ● 1.5 μg/kg fentanyl diluted to 2 mL of NS ● Bolus: 1 mg/kg propofol, 1.5 μg/kg fentanyl ● Infusion of 50 μg/kg/min	92	3–14 years	Reduction of fracture, I&D abscess, wound debridement	1. Recovery time 2. Adverse effects 3. Hemodynamic parameters
Shah 2011 [17]	Ketofol vs. ketamine	● 0.5 mg/kg ketamine + 0.5 mg/kg propofol ● Additional 0.5 mg/kg propofol, if needed	● 1 mg/kg ketamine + intralipid placebo ● Additional 0.25 mg/kg ketamine, if needed	140	Median (IQR): 11 (7–14) years	Closed manual reduction	1. Recovery time 2. Adverse events 3. Satisfaction 4. Hemodynamic parameters
Schmitz 2018 [15]	Ketofol vs. propofol	● 1 mg/kg ketamine (5%) + 0.5 mg/kg propofol (1%) + 0.03 mL/kg NS ● 5 mg/kg/h propofol infusion	● 1 mg/kg propofol (1%) bolus ● 10 mg/kg/h propofol infusion	351	3 months to 10 years	Elective MRI	1. Recovery time 2. Satisfaction 3. Adverse events 4. Hemodynamic parameters
Tewari 2018 [21]	Ketofol vs. dexmedetomidine-propofol	● Bolus: 1 mg/kg ketamine + 2 mg/kg propofol over 10 min ● Infusion: 0.5 mg/kg/h ketamine and 4–6 mg/kg/h propofol	● Bolus: 1 μg/kg dexmedetomidine and 2 mg/kg propofol over 10 min ● Infusion: 0.25–0.75 μg/kg/h dexmedetomidine and 4–6 mg/kg/h propofol	56	7–16 years	Congenital acyanotic heart disease considered amenable for device closure	1. Recovery time 2. Adverse effects
Tosun 2007 [16]	Ketofol vs. propofol-fentanyl	● 0.2 mg/kg ketamine + 1.2 mg/kg propofol ● Additional 0.5–1 mg/kg propofol, if needed	● 0.2 μg/kg fentanyl + 1.2 μg/kg propofol ● Additional 0.5–1 mg/kg propofol, if needed	90	1–16 years	Upper gastrointestinal endoscope	1. Recovery time 2. Adverse effects 3. Hemodynamic parameters
Ulgey 2014 [22]	Ketofol vs. dexmedetomidine- propofol	● 1 mg/kg ketamine + 1 mg/kg propofol ● Maintenance: 1 mg/kg/h ketamine and 100 μg/kg/min propofol ● Additional 0.5 mg/kg propofol, if needed	● 1 μg/kg dexmedetomidine for 5 min, 1 mg/kg propofol ● Maintenance: 0.5 μg/kg/h dexmedetomidine and 100 μg/kg/min propofol ● Additional 0.5 mg/kg propofol, if needed	46	3–14 years	Atrial septal defect for transcatheter closure	1. Recovery time 2. Adverse effects 3. Hemodynamic parameters
Weisz 2017 [18]	Ketofol vs. ketamine	● 0.5 mg/kg ketamine and 0.5 mg/kg propofol ● 3 maximum additional doses of 0.25 mg/kg ketamine and 0.25 mg/kg propofol, if needed	● 1 mg/kg ketamine ● 3 maximum additional doses of 0.5 mg/kg ketamine	183	Ketofol: mean (SD): 9.3 (5) Ketamine: mean (SD): 8.3 (6)	Fracture of dislocation reduction	1. Recovery time 2. Satisfaction 3. Adverse effects
Yalcin 2018 [23]	Ketofol vs. ketamine vs. propofol	● Ratio 1:1, 200 mg propofol (20 mL) + 200 mg ketamine (4 mL) ● 0.6 mg/kg bolus followed by 40–60 μg/kg/min infusion	● 4 mL ketamine diluted with NS to 20 mL, 1 mg/kg bolus followed by 50–60 μg/kg/min ● 2 mg/kg propofol bolus followed by 70–90 μg/kg/min infusion	75	6–12 years	Dental treatment	1. Recovery time 2. Adverse effects 3. Hemodynamic parameters

Of the 11 trials, five trials compared with a single-agent [13, 15, 17, 18, 23] and six trials compared with a combination of agents [14, 16, 19–22]. In all the trials, PSA was administered by experienced practitioners. Among the studies involving single-agent comparison, three trials compared with propofol [13, 15, 23] and three trials compared with ketamine [17, 18, 23]. Among the other six studies, two trials compared with ketamine-dexmedetomidine [19, 20], two trials compared with propofol-dexmedetomidine [21, 22], and two trials compared with propofol-fentanyl [14, 16]. Five trials involved bolus sedation without maintenance infusion [13, 16–19], while six trials involved bolus sedation with maintenance infusion [14, 15, 20–23].

Regarding the ratios of ketamine and propofol, six trials used a 1:1 ratio [17–20, 22, 23], two trials used 1:2 ratio [14, 21], one trial used 2:1 ratio [15], one trial used 1:4 ratio [13], and one trial used 1:6 ratio [16].

**Fig. 2:**
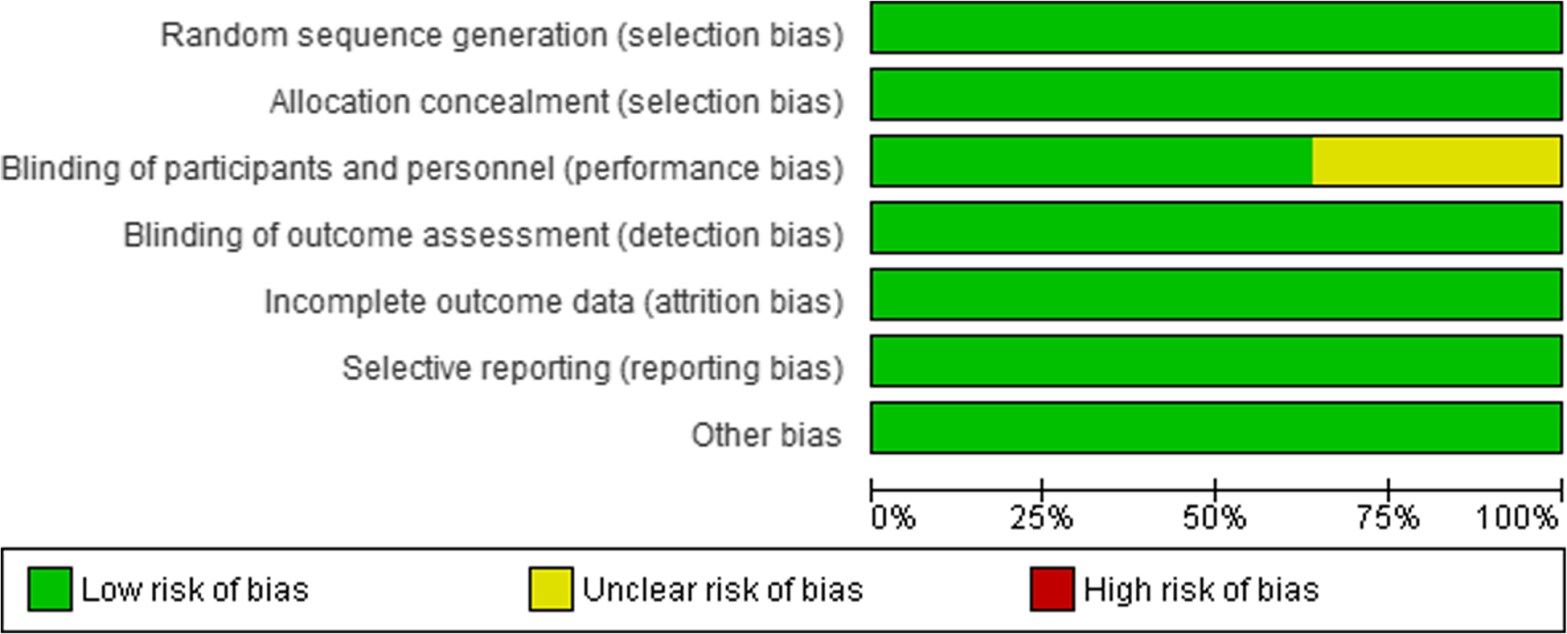
Risk of bias graph: Review of authors’ judgments about each risk of bias item presented as percentage across all included studies

**Fig. 3:**
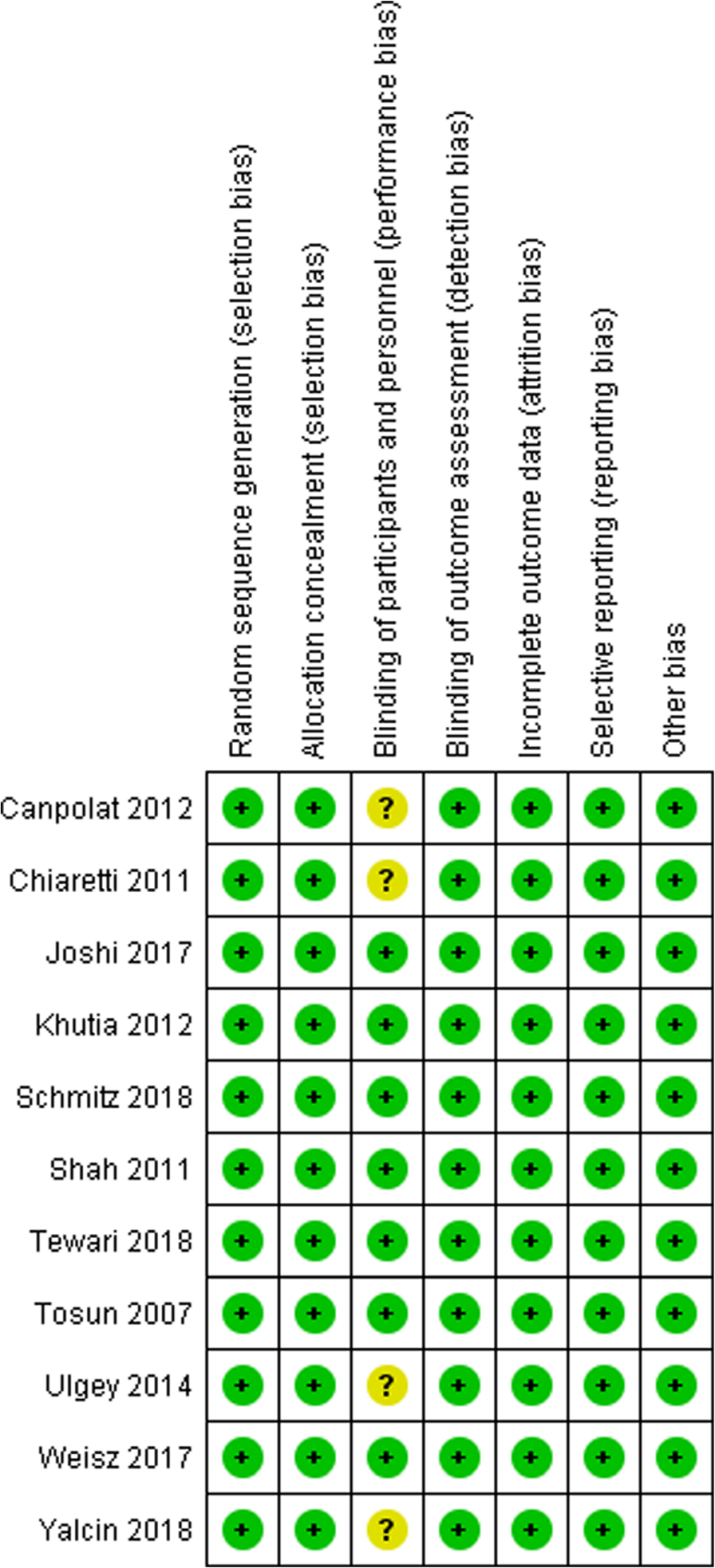
Risk of bias summary: Review of authors’ judgments about each risk of bias item for each included study

The assessment of the risk of bias is shown in Figs. 2 and 3. Figure 2 shows the proportion of studies with low, high, or unclear risk of bias for each risk of bias domain. Figure 3 shows the risk of bias summary for individual studies. All the trials described the method of randomisation used. For randomisation of participants, five trials used the closed envelope method [14, 16, 20, 22, 23]; four trials used the computer-generated method [13, 17, 18, 21]; one trial used the coin toss method [19]; one trial used the 1:1 block randomisation with different strata based on the age and type of magnetic resonance imaging (MRI) [15]. Blinding of participants and personnel was not described in four trials [13, 19, 22, 23]. Six trials analysed all the samples without any withdrawal [16, 19–23]. Five trials carried the intention to treat an analysis in which the participants were analysed according to the group that they were initially assigned [13–15, 17, 18]. All trials reported the outcomes as specified in their respective Methods sections. We detected no other potential source of bias.

A total of 11 trials measured the primary outcome, i.e., recovery time [13–23]. One study defined recovery time as time, in minutes, required for the patient to be conscious and responding to verbal stimuli, airway recovery as the return of gag reflex or cough, and motor recovery as a purposeful movement of limbs [21]; another study defined recovery time as the period needed by the patient to regain consciousness spontaneously [13]; four studies used Steward Recovery Score to define the recovery time but with different cut off points, of which two studies used score of 6 [20, 22] while another two studies used score of 7 [16, 19]; one study defined time from discontinuation of infusion and achievement of Ramsey Sedation Score of 3 as recovery time [14]; another study used Modified Vancouver Sedation Recovery Scale to determine the recovery time [23].

Three trials measured the satisfaction of clinicians [17, 19, 23]. Nine trials measured the secondary outcome, i.e., adverse effects [13–19, 21, 23]. Our protocol intended to report hemodynamic parameters as a secondary outcome. These were not analysed because they either involved comparison in a different unit or a non-comparable group [13–15, 23].. Four trials were not included in the hemodynamic status data because their data were demonstrated in the graph [16, 19, 20, 22].

### Comparison of ketofol vs. single-agent control

**Fig. 4:**
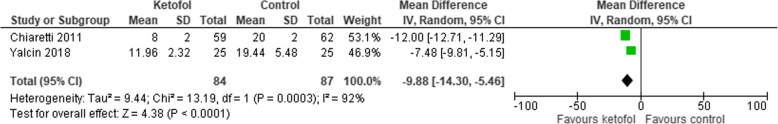
Comparison of ketofol vs. single-agent control with respect to recovery time

**Fig. 5:**
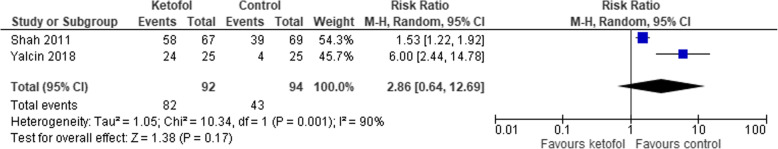
Comparison of ketofol vs. single-agent control with respect to clinician satisfaction

**Fig. 6:**
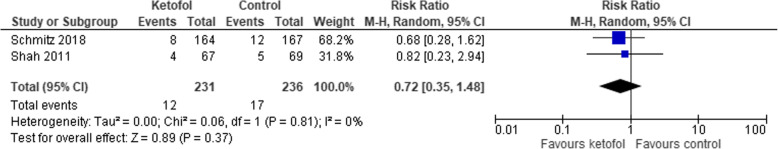
Comparison of ketofol vs. single-agent control with respect to airway obstruction

**Fig. 7:**
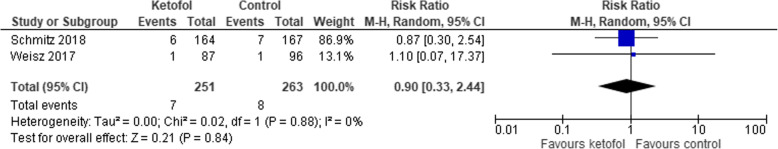
Comparison of ketofol vs. single-agent control with respect to apnoea

**Fig. 8:**
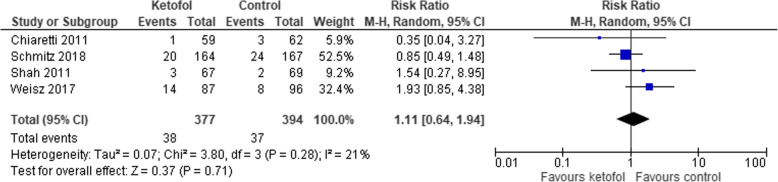
Comparison of ketofol vs. single-agent control with respect to desaturation

**Fig. 9:**
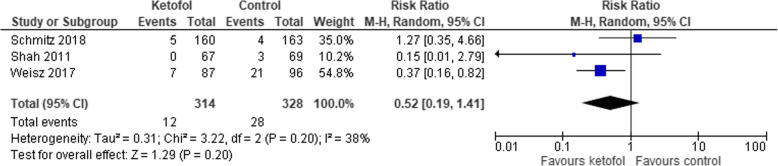
Comparison of ketofol vs. single-agent control with respect to nausea

**Fig. 10:**
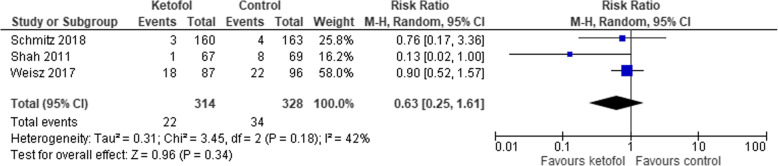
Comparison of ketofol vs. single-agent control with respect to vomiting

Five trials with single agent in the control group were analysed [13, 15, 17, 18, 23]. All five trials measured recovery time; however, three trials reported the results in median and interquartile range (IQR) [15, 17, 18]. Ketofol shows significant effect on recovery time compared to control (MD -9.88, 95% CI: − 14.30 to − 5.46; *P* < 0.001; I^2^ = 92%; 2 trials, 171 participants; low certainty evidence) [13, 23] (Fig. 4, Table 2). However, compared to the control group, the ketofol group showed no difference in terms of clinician satisfaction (RR 2.86, 95% CI: 0.64 to 12.69; *P* = 0.001; I^2^ = 90%; 2 trials, 125 participants; low certainty evidence) [17, 23] (Fig. 5, Table 2); airway obstruction (RR 0.72, 95% CI: 0.35 to 11.48; *P* = 0.810; I^2^ = 0%; 2 trials, 467 participants; high certainty evidence) [15, 17] (Fig. 6, Table 2); apnoea (RR 0.9, 95% CI: 0.33 to 2.44; *P* = 0.880; I^2^ = 0%; 2 trials, 514 participants; high certainty evidence) [15, 18] (Fig. 7, Table 2); desaturation (RR 1.11, 95% CI: 0.64 to 1.94; *P* = 0.280; I^2^ = 21%; 4 trials, 771 participants; high certainty evidence) [13, 15, 17, 18] (Fig. 8, Table 2); nausea (RR 0.52, 95% CI: 0.91 to 1.41; *P* = 0.200; I^2^ = 38%; 3 trials, 642 participants; high certainty evidence) (Fig. 9, Table 2); and vomiting (RR 0.63, 95% CI: 0.25 to 1.61; *P* = 0.180; I^2^ = 42%; 3 trials, 642 participants; high certainty evidence) (Fig. 10, Table 2).

**Table 2:** Summary of Findings for Comparison between Ketofol and Single-Agent

Ketofol compared to a single-agent for procedural sedation and analgesia					
Patient or population: Procedural sedation and analgesia Intervention: Ketofol Comparison: Single agent					
Outcomes	Anticipated absolute effects^*^ (95% CI)		Relative effect (95% CI)	No. of participants (studies)	Certainty of the evidence (GRADE)
	Risk with single agent	Risk with ketofol			
Recovery time	The mean recovery time was 0	MD 9.88 lower (14.3 lower to 5.46 lower)	–	171 (2 RCTs)	⊕ ⊕ ⊝⊝ LOW ^a b^
Satisfaction of clinician	Study population		RR 2.86 (0.64 to 12.69)	186 (2 RCTs)	⊕ ⊕ ⊝⊝ LOW ^b c^
	457 per 1000	1000 per 1000 (293 to 1000)			
Airway obstruction	Study population		RR 0.72 (0.35 to 1.48)	467 (2 RCTs)	⊕ ⊕ ⊕ ⊕ HIGH
	72 per 1000	52 per 1000 (25 to 107)			
Apnea	Study population		RR 0.90 (0.33 to 2.44)	514 (2 RCTs)	⊕ ⊕ ⊕ ⊕ HIGH
	30 per 1000	27 per 1000 (10 to 74)			
Desaturation	Study population		RR 1.11 (0.64 to 1.94)	771 (4 RCTs)	⊕ ⊕ ⊕ ⊕ HIGH
	94 per 1000	104 per 1000 (60 to 182)			
Nausea	Study population		RR 0.52 (0.19 to 1.41)	642 (3 RCTs)	⊕ ⊕ ⊕ ⊕ HIGH
	85 per 1000	44 per 1000 (16 to 120)			
Vomiting	Study population		RR 0.63 (0.25 to 1.61)	642 (3 RCTs)	⊕ ⊕ ⊕ ⊕ HIGH
	104 per 1000	65 per 1000 (26 to 167)			
*The risk in the intervention group (and its 95% confidence interval) is based on the assumed risk in the comparison group and the relative effect of the intervention (and its 95% CI). **CI:** Confidence interval; **RR:** Risk ratio.					
**GRADE (Working Group grades of evidence)** **High certainty:** We are very confident that the true effect lies close to that of the estimate of the effect. **Moderate certainty:** We are moderately confident in the effect estimate; the true effect is likely to be close to the estimate of the effect, but there is a possibility that it is substantially different **Low certainty:** Our confidence in the effect estimate is limited; the true effect may be substantially different from the estimate of the effect. **Very low certainty:** We have very little confidence in the effect estimate; the true effect is likely to be substantially different from the estimate of effect.					

Footnote:

^a^ Duration of treatment varies following different procedures, and thus, required different doses of intervention

^b^ Small sample size

^c^ Subjective outcome with different types of population

### Comparison of Ketofol vs. combined agent control

**Fig. 11:**
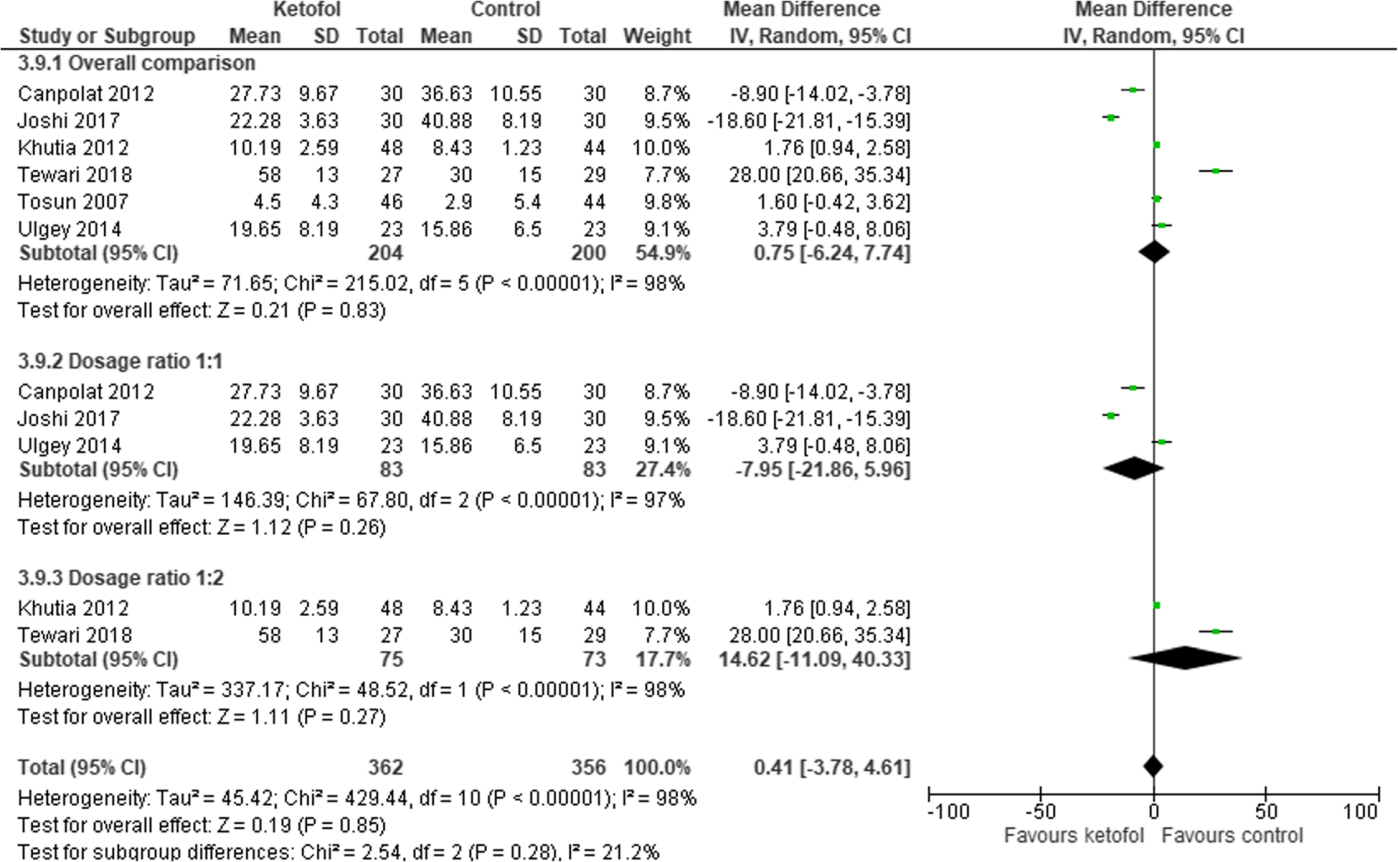
Comparison of ketofol vs. combined agents control with respect to recovery time

**Fig. 12:**
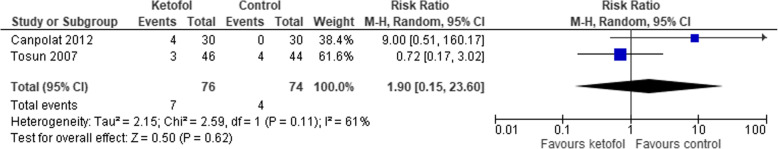
Comparison of ketofol vs. combined agents control with respect to desaturation

**Fig. 13:**
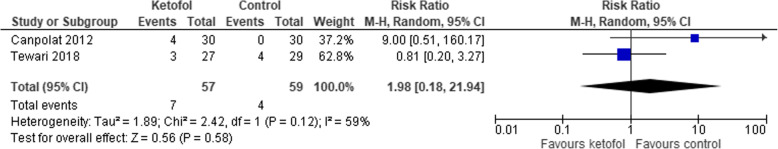
Comparison of ketofol vs. combined agents control with respect to respiratory depression

**Fig. 14:**
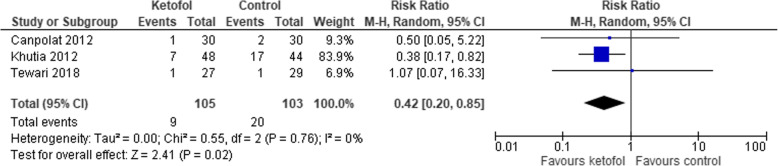
Comparison of ketofol vs. combined agents control with respect to hypotension

**Fig. 15:**
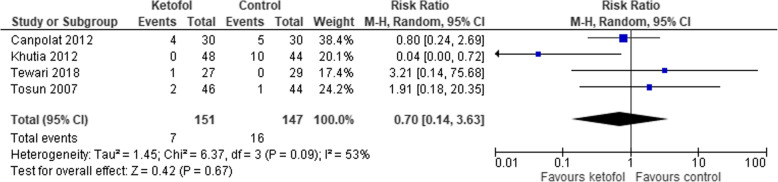
Comparison of ketofol vs. combined agents control with respect to bradycardia

Six trials with combined agent in the control group were analysed [14, 16, 19–22]. Ketofol shows no significant effect on recovery time compared to control (RR 0.75, 95% CI: − 6.24 to 7.74; *P* < 0.001; I^2^ = 98%; 6 trials, 404 participants; moderate certainty evidence) [14, 16, 19–22] (Fig. 11, Table 3). Different RCTs used different ratios of ketamine and propofol in combined agent. We included three studies that used 1:1 ratio [19, 20, 22] and two studies that used the ratio 1:2 [14, 21] in subgroup analysis. With dosage ratio of 1:1, ketofol showed no effect on recovery time compared to control (RR -7.95, 95% CI: − 21.86 to 5.96; *P* < 0.001; I^2^ = 97%; 3 trials, 166 participants; low certainty evidence) (Fig. 11, Table 3). With dosage ratio of 1:2, ketofol also showed no effect on recovery time compared to control (RR 14.62, 95% CI: − 11.09 to 40.33; *P* < 0.001; I^2^ = 98%; 2 trials, 148 participants; low certainty evidence) (Fig. 11, Table 3). With respect to adverse events, compared to control, ketofol showed no effect on desaturation (RR 1.9, 95% CI: 0.15 to 23.6; *P* = 0.110; I^2^ = 61%; 2 trials, 150 participants; low certainty evidence) [16, 19] (Fig. 12, Table 3) and respiratory depression (RR 1.98, 95% CI: 0.18 to 21.94; *P* = 0.120; I^2^ = 59%; 2 trials, 116 participants; low certainty evidence) (Fig. 13, Table 3). However, ketofol showed significant effect on hypotension (RR 4.2, 95% CI: 0.2 to 0.85; *P* = 0.760; I^2^ = 0%; 3 trials, 208 participants; moderate certainty evidence) [14, 19, 21] (Fig. 14, Table 3) but no effect on bradycardia compared to control (RR 0.70, 95% CI: 0.14 to 03.63; *P* = 0.09; I^2^ = 53%; 4 trials, 298 participants; low certainty evidence) [14, 16, 19, 21] (Fig. 15, Table 3).

**Table 3:** Summary of Findings for Comparison between Ketofol and Combined Agents

Ketofol compared to a combined agent for procedural sedation and analgesia					
Patient or population: Procedural Sedation and Analgesia Intervention: Ketofol Comparison: Combined agent					
Outcomes	Anticipated absolute effects^*^ (95% CI)		Relative effect (95% CI)	No. of participants (studies)	Certainty of the evidence (GRADE)
	Risk with combined agent	Risk with ketofol			
Recovery time	The mean recovery time was 0	MD 0.75 higher (6.24 lower to 7.74 higher)	–	404 (6 RCTs)	⊕ ⊕ ⊕⊝ MODERATE ^a^
Recovery time - Dosage ratio 1:1	The mean recovery time - Dosage ratio 1:1 was 0	MD 7.95 lower (21.86 lower to 5.96 higher)	–	166 (3 RCTs)	⊕ ⊕ ⊝⊝ LOW ^b c^
Recovery time - Dosage ratio 1:2	The mean recovery time - Dosage ratio 1:2 was 0	MD 14.62 higher (11.09 lower to 40.33 higher)	–	148 (2 RCTs)	⊕ ⊕ ⊝⊝ LOW ^b c^
Desaturation	Study population		RR 1.90 (0.15 to 23.60)	150 (2 RCTs)	⊕ ⊕ ⊝⊝ LOW ^a c^
	54 per 1000	103 per 1000 (8 to 1000)			
Respiratory depression	Study population		RR 1.98 (0.18 to 21.94)	116 (2 RCTs)	⊕ ⊕ ⊝⊝ LOW ^a c^
	68 per 1000	134 per 1000 (12 to 1000)			
Hypotension	Study population		RR 0.42 (0.20 to 0.85)	208 (3 RCTs)	⊕ ⊕ ⊕⊝ MODERATE ^c^
	194 per 1000	82 per 1000 (39 to 165)			
Bradycardia	Study population		RR 0.70 (0.14 to 3.63)	298 (4 RCTs)	⊕ ⊕ ⊝⊝ LOW ^a c^
	109 per 1000	76 per 1000 (15 to 395)			
*The risk in the intervention group (and its 95% confidence interval) is based on the assumed risk in the comparison group and the **relative effect** of the intervention (and its 95% CI). **CI:** Confidence interval; **MD:** Mean difference; **RR:** Risk ratio**.**					
**GRADE (Working Group grades of evidence)** **High certainty:** We are very confident that the true effect lies close to that of the estimate of the effect. **Moderate certainty:** We are moderately confident in the effect estimate; the true effect is likely to be close to the estimate of the effect, but there is a possibility that it is substantially different. **Low certainty:** Our confidence in the effect estimate is limited; the true effect may be substantially different from the estimate of the effect **Very low certainty:** We have very little confidence in the effect estimate; the true effect is likely to be substantially different from the estimate of effect.					

Footnote:

^a^ Results show large heterogeneity that can be due to the following: 1) different populations: some procedures required a longer duration of treatment, thus, larger doses are required. 2) different ratios of the mixture and dosages of the combination

^b^ Results show large heterogeneity that can be due to different populations and durations of procedures that determine the required dose of intervention

^c^ Small sample size

## Discussion

The concept of combination of ketamine and propofol is based on the synergistic effects and the benefits they provide while countering each-other’s side effects. This review was designed to include all RCTs addressing the effectiveness of ketofol in PSA in the paediatric population compared to other analgesic agents. Ketofol showed a significant effect on recovery time compared to single-agent but showed no differences when compared to a combined agent. The subgroup analyses (different ratios of dosages) showed no differences. However, three studies with small sample sizes in single-agent analyses were not included in our analysis due to a non-usable format that discouraged the generation of an appropriate and meaningful conclusion.

Ketofol showed no effect on clinician satisfaction and respiratory adverse events (airway obstruction, apnoea, desaturation, and respiratory depression) in comparisons with both single and combined agents. In adverse cardiovascular events, ketofol reduced the frequency of hypotension but showed no effect on bradycardia. There was no significant difference between the gastrointestinal adverse events (nausea and vomiting) of ketofol and control groups.

The quality of trial evidence was variable. Generally, there was a low or unclear risk of bias for most trials in most domains. During single-agent comparison, the certainty of the evidence for recovery time was low. We downgraded for inconsistency and imprecision as we noted substantial statistical heterogeneity and small sample size. During combined agent comparison, the certainty of the evidence for recovery time was moderate. We downgraded because of apparent inconsistencies. The quality of the evidence for both subgroup analyses for dosage ratios 1:1 and 1:2 was downgraded two levels due to heterogeneity and small sample size. A low-quality rating was assigned to the pooled estimates of effect for the outcome of clinician satisfaction due to small sample size and large heterogeneity.

For the outcome of adverse events of nausea, vomiting, airway obstruction, apnoea, and desaturation in single-agent comparison, the overall quality of the evidence was judged to be high. However, during combined agent comparison, the quality of the evidence for respiratory depression, desaturation, and bradycardia was judged to be low. The evidence for these outcomes was from small sample size trials, and our certainty in the evidence was reduced owing to the imprecision. We also noted the unexplained difference between study data, and thus, we downgraded for inconsistency. Evidence for hypotension was obtained from a few studies and few participants. Therefore, the evidence was imprecise, and this reduced our certainty in the evidence to moderate (Table 3).

Clinical heterogeneity was anticipated, and therefore, we explored the statistical heterogeneity through subgroup analysis. Specifically, we analysed on the basis of the different ratios of dosages in the mixture where it was best reflecting the interventions present in the analysed data.

Prior to our meta-analysis, there is a lack of comprehensive review regarding the use of ketofol in PSA in the paediatric population. We found a systematic review and meta-analysis that compared ketofol with propofol alone in adults and children, which included six RCTs with a total of 932 patients [24]. In the review, two RCTs were peer-reviewed abstracts selected for presentation at international conferences, one of these RCTs included paediatric patients aged 3 to 18 years old, which was excluded from our study [25] due to the absence of a full-text article. The above-mentioned review only included ED patients undergoing PSA for any painful non-elective procedures; whereas, the current review included both ED patients and those undergoing elective procedures. They concluded a significantly higher frequency of adverse respiratory events in cases involving ketofol compared to those involving propofol. However, our study showed no such difference compared to the control group. Their results with respect to recovery time were inconclusive owing to the non-usable data of the included RCTs.

Another systematic review and meta-analysis that compared ketofol to propofol included all relevant RCTs, regardless of patient’s age, sex, location, publication year, and language [5]. The review reported outcomes of various adverse events but did not describe recovery time. Contrary to our findings, the outcome of respiratory complications that require intervention and bradycardia, was significant compared to the control group. However, the findings related to other outcomes of hypotension, nausea, and vomiting were similar to those obtained in the current review.

## Limitations

This meta-analysis has several limitations. First, our study demonstrates significant heterogeneity for the outcome of recovery time due to multiple factors, including the variety among the patient populations, type and duration of different procedures, a different dosage of bolus and maintenance with a varying method of mixture. Most of the studies did not use standardised and quantifiable endpoints to define recovery time. However, exploration of an effect in subgroup analyses found no difference between them. Second, hemodynamic parameters that play an important role in monitoring patient who undergoes PSA were not analysed due to lack of presentable data with a different unit of measurement.

Additionally, small samples size ranging from 46 to 351 participants discouraged us from generating appropriate and meaningful analyses. Lastly, we conducted a thorough search and used two review authors to assess the study eligibility, extract the data, and assess the risk of bias in the included studies, thereby reducing potential bias in the review process. Although the search strategy used to identify potentially relevant studies was extensive, only the articles in English language were included in this review. Some relevant studies may have been skipped if they were conducted in other languages.

## Conclusion

There is a low certainty evidence that ketofol improves the recovery time compared to single-agent and reduces the frequency of hypotension along with a moderate certainty of evidence for PSA in the paediatric population. There were no differences in other adverse effects during comparison with either single-agent or combined agent. A larger sample size would increase the certainty of this evidence. Our study can be a guide for practitioners to decide the choice of agents in PSA.

Future studies addressing this research question might benefit by focusing on some of the limitations that we encountered with current evidence. Firstly, in order to increase the certainty in effect for most outcomes in this review, larger sample size is required. More research in this field would also enable a more precise exploration of subgroups. In particular, the favourable dosages of bolus and maintenance, ratio, and method of mixture for the different combinations of ketamine and propofol have an essential influence on their effects, such as recovery time, sedation level, and hemodynamic adaptation. Secondly, there is currently a lack of hemodynamic data, which plays a significant role in monitoring the patient. The subtle change of hemodynamic parameters might help to prevent complications during PSA, and plays a vital role in the choices of PSA by the physician. Finally, future studies should clearly define recovery time as well as sedation level using a standardised scoring system.

## Data Availability

The datasets used and/or analysed during the current study are available from the corresponding author on reasonable request.

## Appendix

### Search Strategy

1. (child): ti, ab, kw OR (pediatric): ti, ab, kw
2. (ketofol): ti, ab, kw OR (ketamine-propofol): ti, ab, kw
3. (procedural sedation OR analgesia): ti, ab, kw
4. #1 AND #2 AND #3

## Abbreviations

CENTRAL: Cochrane Central Register of Controlled Trials
CI: Confidence interval
ED: Emergency department
GRADE: Grading of recommendations assessment, development, and evaluation
I&D: Incision and drainage
ICTRP: International clinical trial registry platform
ICU: Intensive care unit
IQR: Interquartile range
IV: Intravenous
Ketofol: Ketamine-propofol
Kg: Kilogram
MD: Mean differences
MEDLINE: Medical Literature Analysis and Retrieval System
Mg: Milligram
Min: Minute
MRI: Magnetic resonance imaging
NS: Normal saline
OR: Odds ratio
OT: Operation theatre
P: *p*-value
PROSPERO: International Prospective Register of Systematic Reviews
PSA: Procedural sedation and analgesia
RCTs: Randomised control trials
RevMan: Review manager
RR: Risk ratio
SD: Standard deviation
USA: United States of America
μg: Microgram
